# A synthetic kisspeptin analog that triggers ovulation and advances puberty

**DOI:** 10.1038/srep26908

**Published:** 2016-06-01

**Authors:** C. Decourt, V. Robert, K. Anger, M. Galibert, J.-B. Madinier, X. Liu, H. Dardente, D. Lomet, A. F. Delmas, A. Caraty, A. E. Herbison, G. M Anderson, V. Aucagne, M. Beltramo

**Affiliations:** 1 UMR Physiologie de la Reproduction et des Comportements (INRA, UMR85; CNRS, UMR7247, Université François Rabelais Tours, IFCE) F-37380 Nouzilly, France; 2 Centre de Biophysique Moléculaire (CNRS UPR 4301) F-45071 Orléans cedex 2, France; 3 Centre for Neuroendocrinology, PO Box 913, University of Otago School of Medical Sciences Dunedin 9054, New Zealand

## Abstract

The neuropeptide kisspeptin and its receptor, KiSS1R, govern the reproductive timeline of mammals by triggering puberty onset and promoting ovulation by stimulating gonadotrophin-releasing hormone (GnRH) secretion. To overcome the drawback of kisspeptin short half-life we designed kisspeptin analogs combining original modifications, triazole peptidomimetic and albumin binding motif, to reduce proteolytic degradation and to slow down renal clearance, respectively. These analogs showed improved *in vitro* potency and dramatically enhanced pharmacodynamics. When injected intramuscularly into ewes (15 nmol/ewe) primed with a progestogen, the best analog (compound **6**, **C6**) induced synchronized ovulations in both breeding and non-breeding seasons. Ovulations were fertile as demonstrated by the delivery of lambs at term. **C6** was also fully active in both female and male mice but was completely inactive in KiSS1R KO mice. Electrophysiological recordings of GnRH neurons from brain slices of GnRH-GFP mice indicated that **C6** exerted a direct excitatory action on GnRH neurons. Finally, in prepubertal female mice daily injections (0.3 nmol/mouse) for five days significantly advanced puberty. **C6** ability to trigger ovulation and advance puberty demonstrates that kisspeptin analogs may find application in the management of livestock reproduction and opens new possibilities for the treatment of reproductive disorders in humans.

Suboptimal reproduction in livestock and reproductive disorders in humans are problems that typically share a common cause: an inadequate activity of the hypothalamus-pituitary axis leading to an insufficient secretion of gonadotropins (luteinizing hormone, LH, and follicle stimulating hormone, FSH). The hypothalamic neuropeptide kisspeptin (Kp) potently stimulates gonadotropin release by triggering the secretion of GnRH (gonadotropin releasing hormone)^1^.

Several forms of endogenous Kp (named according to their length in amino acids Kp54, Kp14, Kp13, and Kp10) have been described^1^. Two of them, Kp10 and Kp54, were used to probe Kp potential applications. In ewes during the non-breeding season, a condition characterized by low levels of circulating gonadotropins and therefore resembling gonadotropic deficiency observed in some human pathology, a continuous perfusion of Kp10 induced ovulation^2^. This is in line with the restoration of gonadotropin pulsatility following continuous Kp10 infusion in patients affected by hypogonadotropic hypogonadism (HH), induced by loss-of-function mutation in neurokinin B (NKB) or its receptor (TAC3R)^3^. In addition in women affected by hypothalamic amenorrhea Kp54 injections had positive effect stimulating an increase of LH^4^,^5^. Furthermore, in juvenile monkeys (*Macaca mulatta*) repeated hourly administration of Kp10 over a 48 hours period triggered LH and FSH release suggesting that the transition from juvenile to pubertal state is controlled by KiSS1R activation^6^. Further supporting this observation chronic central administration of Kp10 in female rats induced an advanced vaginal opening^7^. These observations support the concept that the Kp system is a key lever to control reproduction and is a therapeutic target.

However, the use of the endogenous peptides has clear limits. Desired effects were achieved only by perfusion (intravenous or intracerebroventricular) or by hourly injection. These dosing regimens are unsuitable for livestock application and cumbersome for human therapy. In human intracerebroventricular administration of drugs is normally not an option, and intravenous infusion or multiple intravenous injections requires hospitalization, medical monitoring and are normally subject to low patient compliance. On the other hand, the use of intracerebroventricular or intravenous perfusion in a farming setting is not even conceivable and multiple intravenous injections would require veterinary skills resulting in an unacceptable cost increase. The design of molecules with improved pharmacological profile, capable of producing the same effect after a single intramuscular injection, would represent a key advance towards clinical and field applications.

At present only two groups have performed a significant medicinal chemistry attempt to develop KiSS1R agonists with improved *in vivo* pharmacological profile compared to Kp. The first group produced a series of decapeptide analogs of Kp10 in which a single amino acid was exchanged with an enantiomer or with another amino acid and the best analog was tested *in vivo* for its capacity to stimulate the gonadotropic axis^8^. The second group performed multiples modifications of the Kp10 template resulting in several degradation resistant analogs. The aim of that research was to curtail GnRH secretion by desensitizing KiSS1R with an extremely long-lived agonist, and consequently reduce to castrate level circulating gonadotropins and testosterone^9^,^10^. Nevertheless, depending on the dosing regimen, such type of analogs could also be used to stimulate the reproductive system.

Surprisingly, however, this medicinal chemistry effort did not lead to a significant exploitation of these molecules to evaluate their stimulatory effect on reproductive activity. In our previous work^11^ we designed a series of Kp10 analogs and evaluated their capacity to stimulate the reproductive axis. When tested *in vivo* these analogs showed an enhanced pharmacological profile compared to Kp10, but the increase of LH and FSH secretion lasted only several hours. To be used in a preclinical proof of concept for reproduction, these molecules needed further refinement. In the present work we report the design and profiling of a new potent and selective Kp analog and its successful application to perform a preclinical proof of concept for ovulation stimulation and puberty advancement.

## Results

### New kisspeptin analogs with improved *in vitro* and *in vivo* activity

**Figure 1:**
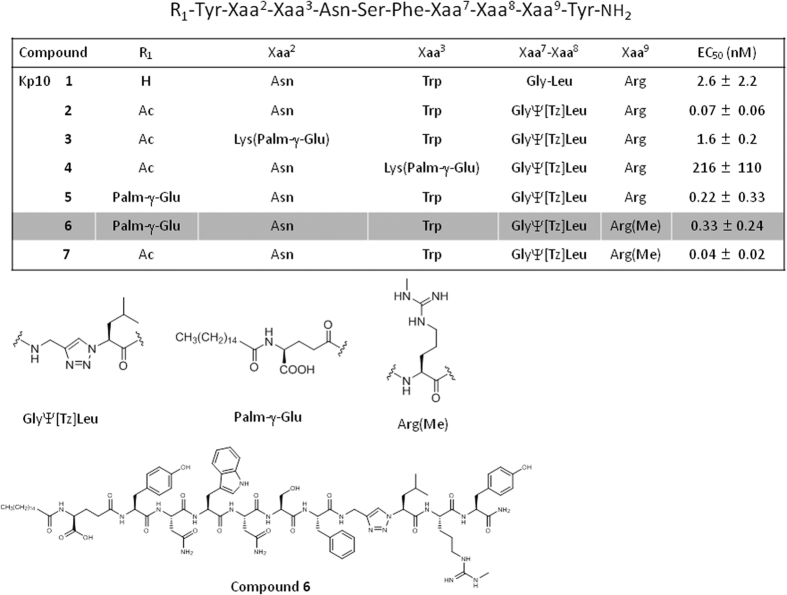
Chemical structures and half maximal effective concentration (mean ± SEM, N = 3) of kisspeptin 10 and analogs C2–C7 as measured in a calcium mobilization assay in KiSS1R-transfected HEK293 cell line. Ac: acetyl; Palm-γ-Glu: *N*-palmitoylated-γ-glutamate, Tz: Triazole; Me: Methyl.

Our previous analogs featured an N-terminal acetyl group and a 1,2,3-triazole heterocycle as a non-hydrolyzable peptide bond mimic to protect it against degradation (compound **2**, **C2**) (Fig. 1). However, **C2** only showed a modest enhancement in *in vivo* duration of action, attributed to fast excretion. Accordingly, we incorporated the serum albumin-binding motif *N*-palmitoylated-γ-glutamate on the side chain of a Lys residue^12^,^13^ inserted in either position 2 or 3 (compounds **3** and **4**, **C3** and **C4**). Despite their reduced potency compared to analog **C2**, the best of the two analogs, **C3**, had an improved *in vivo* activity. In the present work we focused on two additional modifications in order to obtain a molecule suited for a preclinical proof of concept: introduction of the albumin-binding motif on the N-terminal amine of the triazolopeptide in place of the acetyl group of **C2**, rather than on the amine of the side chain of a lysine (compounds **5** and **6**, **C5** and **C6**), and ω-methylation of Arg^9^ (compounds **6** and **7**, **C6** and **C7**). Arg^9^ ω-methylation has been shown to dramatically enhance proteolytic stability of Kp10 in blood serum by conferring resistance to trypsin-like proteases^14^.

We first checked that the introduction of a *N*^ω^-methyl group on Arg^9^ did not significantly modify *in vitro* activity in intracellular Ca^2+^ mobilization assays (Fig. 1, compare **C2** and **C7**). We also found that N-terminal (rather than Lys side chain) introduction of the albumin-binding motif was beneficial, as compound **C5** showed a markedly improved EC_50_ compared to **C3** (Fig. 1). Arg^9^ methylation (**C6**) hardly impacted potency (from 0.22 ± 0.33 nM (**C5**) to 0.33 ± 0.24 nM (**C6**)) (Fig. 1). Hence, **C6** still showed a significant ~5-fold enhancement in EC_50_ compared to our first generation analog **C3**. Accordingly, we anticipated that **C5** and **C6**, associating an albumin binding tag to an increased *in vitro* potency and, in the case of **C6**, an expected enhanced proteolytic resistance, would have significantly improved pharmacodynamics and were selected for further *in vivo* studies.

**Figure 2:**
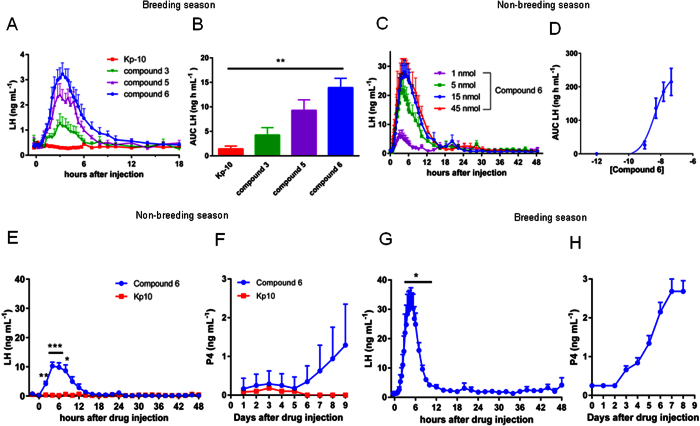
In the ewe C6 increases LH plasma concentration and triggers ovulation during breeding and non-breeding seasons. (**A**)The effect of Kp10, **C3**, **C5**, or **C6** (15 nmol/ewe) were compared during the breeding season in ewes under flugestone acetate (FA) treatment, by measuring LH plasma concentration. During an artificial luteal phase induced by the presence of an intravaginal sponge containing FA, intramuscular injection of Kp10, had no effect on LH basal level, whereas the other compounds increased LH plasma concentration from 6 (**C3**) to more than 10 (**C6**) hours. (**B**) Evaluation of the total amount of LH secreted (AUC) show that **C6** is the most active (Kruskal- Wallis P = 0.0028, followed by Dunn’s multiple comparisons test). (**C–D**) During the breeding season injection of **C6** produced a clear dose-response with an ED_50_ of 4.2 nmol as calculated using the AUC of the different doses. Data are the means ± SEM, N = 6. (**E,F**) Intramuscular administration of 15 nmol/ewe of Kp10 during the non-breeding season, 24 hours after withdrawal of flugestone acetate (FA), had no effect on LH plasma concentration or progesterone level. Conversely, administration of 15 nmol/ewe of **C6**, induced a highly synchronous LH surge in all ewes during both the non-breeding (**E)** and breeding season (**G**). In both non-breeding and breeding season the treatment increases P4 level, over the 1 ng mL^−1^ threshold, indicating the formation of *corpora lutea* (**F**,**H**). Data are the means + SEM, (N = 6 for Kp10 panels E and F, N = 12 for C6 panels E and F and N = 6 for panels G and H). For panel E and G treatment was compared to basal using ANOVA on repeated measure corrected by Geisser-Greenhouse method to compensate for different variance between groups (F = 17.91; P < 0.005), followed by Dunnett’s multiple comparisons test.

Equimolar amounts of Kp10, **C3**, **C5** and **C6** were injected intramuscularly in ewes during an artificial luteal phase induced by treatment with flugestone acetate (FA) during the breeding season. Under this condition Kp10 (15 nmol/ewe) had only a negligible effect on LH plasma concentration (Fig. 2A,B). Conversely, the three analogs induced a clear and prolonged increase in LH with **C6** showing the best profile (Fig. 2A,B). Further testing of **C6** during the non-breeding season produced a clear dose response curve with an ED_50_ of 4.2 nmol (95% CI 1.2–14.0 nmol) (Fig. 2C,D). Based on these results **C6** was used for subsequent studies.

### In the ewe C6 induces synchronous LH surges in both breeding and non-breeding season

Ewes were pretreated with FA for 14 days, to simulate a luteal phase. In the non-breeding season intramuscular injection of Kp10 (15 nmol/ewe) 24 hours after ending of FA treatment, induced no increase of LH plasma concentration (Fig. 2E). Conversely, under the same condition an equimolar amount of the analog produced in all ewes a robust LH increase (peak value 10.3 ± 3.7 ng mL^−1^) reaching a highly synchronous maximum between 4 and 6 hours post-injection (Fig. 2E). Furthermore, 14 hours post-treatment the LH level was still three times higher than the pre-injection concentration (0.4 ± 0.3 ng mL^−1^ and 1.4 ± 1.1 ng mL^−1^ basal and treated respectively).

In the ewes injected with Kp10 progesterone level never reached the threshold of 1 ng mL^−1^ (Fig. 2F) which is considered the hallmark of ovulation and *corpus luteum* formation. Remarkably, in 8 out of 12 treated ewes treated with **C6** progesterone was above the threshold (from 1.1 to 3.1 ng mL^−1^) and remained elevated for several days indicating the formation of viable *corpora lutea* (Fig. 2F). For individual LH and progesterone profiles see Figure S1(A,B) of supplementary material.

During the breeding season **C6** injection, 24 hours after FA withdrawal, led to a highly synchronized increase of LH 5 hours post-injection (Fig. 2G). The fold increase compared to basal was similar in breeding and non-breeding season (24 vs 26 fold respectively) but higher in absolute value during the breeding season (LH 31.3 ± 6.1 ng mL^−1^ vs 10.3 ± 3.7 ng mL^−1^). LH plasma level remained elevated, three times the basal, up to 10 hours post-injection (1.3 ± 0.1 ng mL^−1^ and 4.2 ± 0.5 ng mL^−1^ basal and treated respectively) (Fig. 2G). In all ewes progesterone levels increased and remained elevated up to the end of sampling (Fig. 2H), confirming the formation of *corpora lutea*. For individual LH and progesterone profiles see Figure S1(C,D) of supplementary material. Analysis of FSH showed the induction of a biphasic increase in its plasma concentration reminiscent of that observed during a natural estrous cycle in the ewe (see supplementary Figure S2).

### Ovulations synchronized by C6 are fertile

To evaluate if ovulations were fertile we used the same protocol during the breeding season, and 6 hours after **C6** injection females were individually introduced into a ram’s pen. In the presence of the ram 8 out of 10 ewes showed clear estrus behavior (immobilization with head lowered, looking over the shoulder as the ram nudges her flank or anogenital region) followed by mounting. After overnight exposure to the ram equipped with a marking apron, all ewes were marked suggesting successful servicing. In 7 out of 10 ewes progesterone level remained high up to 19 days post-injection, indicating pregnancy. Transrectal ultrasonography confirmed the presence of viable fetuses in the ewes with high progesterone level and healthy lambs were born at term.

### C6 stimulates GnRH neurons in brain slices of GnRH-GFP mice

**Figure 3:**
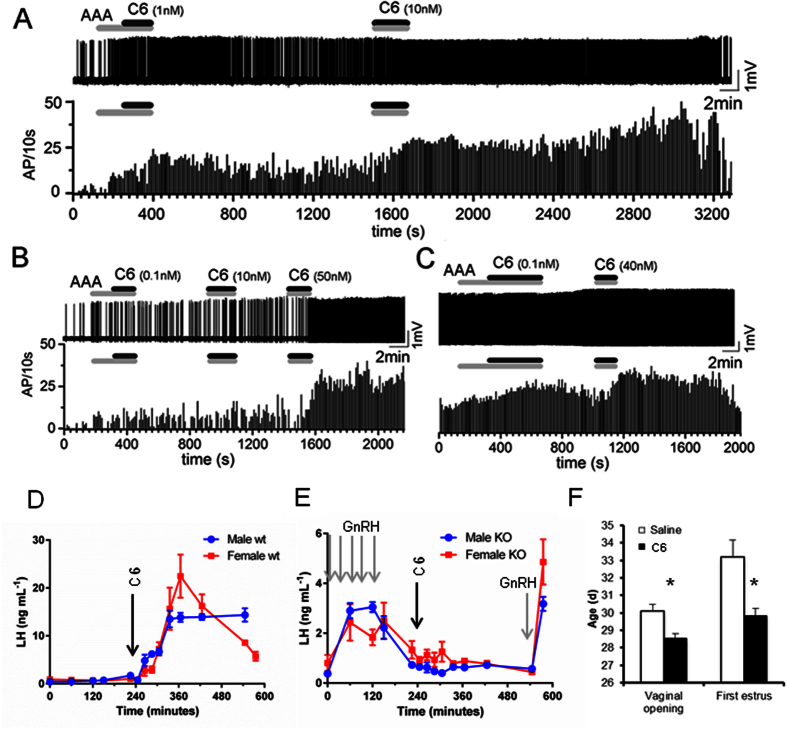
In mice C6 excites GnRH neurons, induces LH secretion by a KiSS1R-dependent mechanism and its repeated administration advances puberty. (**A–C**) Cell-attached voltage recordings of three GnRH neurons from adult female GnRH-GFP mice (with frequency histograms below each voltage recording) showing the dose-dependent activation of GnRH neuron firing by 0.1–40 nM **C6**. A cocktail of amino acid receptor antagonists (AAA, including kynurenic acid 2 mM, GABAzin 5 mM or bicuculline 10 mM) was applied before or with **C6** as indicated by the grey bars. (**D**) Administration of **C6** (arrow) to male or female mice *in vivo* induces a rapid and prolonged increase of LH plasma concentration in males and a transient LH surge-like in females. (**E**) In KiSS1R KO mice, GnRH priming (grey arrows) induces an increase in plasma LH concentrations over basal in both males and females. However, administration of **C6** has no effect. Further stimulation with GnRH (grey arrow) after **C6** application still induces an increase of LH confirming that the pituitary is still responsive. Data are the mean ± SEM, N = 4 for males and N = 7 for females. (**F**) Daily injection of **C6** (0.15 nmol/mouse) from postnatal day 26 to 30 significantly advanced vaginal opening (Mann Whitney test P = 0.0014) and first estrus (Mann Whitney test P = 0.0036). Data are the means ± SEM, N = 10.

To assess if **C6** directly acts on GnRH neurons, the drug at concentrations ranging from 0.01 to 40 nM was applied for 2–4 minutes to preoptic area brain slices prepared from adult female GnRH-GFP mice (n = 29 GnRH neurons tested from 4 diestrus and 2 estrus mice). All recordings were undertaken in the presence of a cocktail of ionotropic GABA and glutamate receptor antagonists (kynurenic acid 2 mM, GABAzine 5 mM or bicuculline 10 mM) to remove the possibility of indirect actions of **C6** upon GnRH neurons. Cell-attached voltage recordings revealed that **C6** exerted a potent dose-dependent excitatory action on the firing rate of individual GnRH neurons (Fig. 3A–C). Approximately 50% of GnRH neurons were activated across the concentration range (0.01 nM, 5/10 cells; 0.1 nM, 2/6 cells; 1 nM, 9/18 cells; 10 nM, 6/14 cells; 40 nM 3/7 cells).

### C6 increases LH plasma concentration in male and female mice

Intraperitoneal injection of **C6** (0.3 nmol/mouse) to wild type male mice resulted in an extremely rapid increase from a basal circulating LH concentration of 0.8 ± 0.4 ng mL^−1^ to a plateau of 13.9 ± 1.8 ng mL^−1^ at about 90 minutes post-injection. This plateau concentration lasted until experiment end (Fig. 3D). In wild type diestrus females **C6** produced a sharp increase of LH from basal concentrations of 0.7 ± 0.3 ng mL^−1^ to a peak of >20 ng mL^−1^ about 2 hours post-injection followed by a rapid decline to 5–6 ng mL^−1^ by experiment end (Fig. 3D).

### C6 has no effect on LH secretion in KiSS1R KO mice

*In vitro* calcium mobilization assay performed on HEK293 cell line transfected with KiSS1R and on non-transfected parental cell line clearly indicated that in the absence of KiSS1R **C6** has no effect (see supplementary Figure S3). To establish if the increase in LH triggered by **C6** is mediated by KiSS1R we performed a set of experiments in KiSS1R KO mice. Basal LH levels in KiSS1R KO mice were 0.4 ± 0.1 ng mL^−1^ and 0.8 ± 0.4 ng mL^−1^ in males and diestrus females respectively. Priming with GnRH resulted in a 3–6 fold increase of circulating LH concentration at 15 minutes post injection (2.7 ± 0.6 ng mL^−1^ and 2.3 ± 0.8 ng mL^−1^ in males and females respectively) that declined to basal level 2 hours after the last GnRH injection (Fig. 3E). Administration of 0.3 nmol of **C6** had no effect on LH concentration up to 5 hours post-injection. Circulating LH concentrations over this time averaged 0.6 ± 0.1 ng mL^−1^ and 0.9 ± 0.5 ng mL^−1^ in male and female mice respectively (Fig. 3E). A further injection of GnRH at the end of experiment produced again a rise in LH concentration (3.2 ± 0.4 ng mL^−1^ 4.8 ± 1.9 ng mL^−1^ in males and females respectively) confirming the functionality of the pituitary gland.

### C6 advances the age of puberty in mice

Daily injection of **C6** (0.15 nmol/mouse) from postnatal day 26 to 30 advanced vaginal opening in treated animals compared to controls (28.5 ± 0.2 vs 30.1 ± 0.4 days respectively (Mann Whitney test P = 0.0014) (Fig. 3F). All animals injected with **C6** showed vaginal opening by day 29 versus day 32 for control animals (supplementary Figure S4A). Consistent with this, first estrus was detected much earlier in treated animals compared to controls (29.8 ± 0.4 vs 33.2 ± 1.0 days respectively, Mann Whitney test P = 0.0036) (Fig. 3F and supplementary Figure S4B). On day 26 in most control animals LH level were below the detection limit of the assay and slightly increased by day 30. The first and last **C6** injections increased LH level, but on day 30 the increase was significantly lower than on day 26 (see supplementary Figure S5).

## Discussion

The present work discloses the synthesis of a Kp10 analog (**C6**) capable, following progestogen priming, of inducing fertile ovulation in ewes after a single intramuscular injection. **C6** also advanced puberty in female mice. These results imply that Kp analogs hold potential for both the management of livestock reproduction and the treatment of reproductive disorders.

We have recently described a triazole-containing lipopseudopeptide with an improved pharmacodynamics profile compared to Kp10^11^(C3). Nevertheless, further improvement of its profile was required to perform a preclinical proof of concept. Here, two new compounds (**C5** and **C6**) are described, combining an N-terminal albumin-binding motif, a Gly^6^Ψ[Tz]Leu^7^ non-hydrolyzable peptide bond mimics, and an *N*^ω^-methylation on Arg^9^ in the case of **C6**. Introduction of the albumin-binding motif on the N-terminus of the peptide rather than on the side chain of a lysine largely improved EC_50_, while simplifying the synthetic processes: indeed, solid-phase peptide synthesis of a branched structure typically requires an orthogonal protection scheme and non-standard synthetic steps. Note that, to our knowledge, the present work constitutes the first example of introduction of an *N*-palmitoylated glutamate N-terminally rather than as a lysine substituent, that could be of general interest for other peptide-based drugs.

To check if the improved *in vitro* potencies of **C5** and **C6** were mirrored by improved *in vivo* activities, their activities were compared to that of Kp10 and the previously published **C3**. Remarkably, both compounds were extremely active at increasing LH plasma concentration under conditions where a single injection of Kp10 had no effect. Reflecting the increased resistance to proteolysis expected from Arg^9^ methylation, **C6** induced a significantly longer and higher increase of LH compared to **C5**. In addition, compared to **C3**, the effect of **C6** was more than twice larger in amplitude and nearly twice longer in duration confirming a substantial pharmacodynamics improvement. Notably, this effect was obtained after intramuscular injection overcoming the need for intravenous or intracerebral delivery; this represents an important advance towards potential applications.

A previous study reported the synthesis of a Kp10 analog in which the N-terminal amino acid [Y] was exchanged with its enantiomer [dY]. This modification probably reduced the N-terminal degradation by exopeptidases. When injected intraperitoneally in mice this analog was superior to Kp10. Nevertheless, its effect on LH was already declining 60 minutes post-injection and disappeared after 120 minutes^8^.

However, the best Kp analogs described in the literature are two nonapeptides: TAK-683 and TAK-448. Both compounds were active after a single subcutaneous injection and capable of inducing a rapid and long lasting increase of LH in healthy men. However, no clear dose-response relationship was obtained with either compound^10^,^15^. This is a serious drawback when tailoring the dose to the effect. In this respect the clear dose-dependent action of **C6** represents a significant advantage. We have previously reported that the azaGly^6^ modification used in the TAK compounds is less efficient in preventing degradation of the Phe^5^Gly^6^Leu^7^ sequence that the Gly^6^Ψ[Tz]Leu^7^ used in our analog^11^. Nevertheless, it seems unlikely that this could account for the difference in the dose-dependency observed between **C6** and TAK-683/TAK-448. Considering that no modification to reduce renal clearance was incorporated in TAK-683 and TAK-448, it is plausible that the combination of the Gly^6^Ψ[Tz]Leu^7^ and the albumin binding motif accounts for the improved profile.

In seasonal breeding livestock (e.g. sheep and goats) induction of ovulation in the non-breeding season is required to guarantee product availability throughout the year and maintain economic viability^16^. In humans, ovulation induction is a therapeutic goal, in this case to treat forms of HH such as hypothalamic amenorrhea. In addition, the possibility to trigger appropriately timed puberty is desirable to improve livestock utilization and is a medical requirement in patients affected by delayed puberty. To assess if **C6** may be useful to address these unmet needs we tested its effects in two model species. To perform a proof of concept for ovulation induction/synchronization, the ewe has the advantage of being a species subjected to intensive commercial reproduction management. In addition, during the non-breeding season the hypothalamo-pituitary-gonadal (HPG) axis of the ewe shows a reduced activity resembling that observed in HH. Results obtained during this period may give hints on the possibility to treat various types of reproductive disorders.

In the non-breeding season, following progestogen priming, a single intramuscular administration of **C6** was able to induce LH and FSH surges followed by *corpora lutea* formation, a hallmark of ovulation. During the breeding season **C6** triggered perfectly synchronized ovulations that were fertile as shown by delivery of healthy lambs. Noteworthy, TAK-683 was recently tested for its capacity to stimulate an LH surge in goats. Depending on the phase of the estrus cycle, acute administration resulted either in a stimulatory effect capable of triggering an LH surge^17^, or in a perturbation of follicular maturation, reduction of ovulating follicle size and reduced progesterone level during the ensuing luteal phase^18^. At this time it is unclear if TAK-683 would be suitable for induction and/or synchronization of ovulations. In addition there is no information about the fertility of ovulations resulting from this treatment. Another kisspeptin analog (FTM080) was recently tested by intravenous injection in anestrus ewe. FTM080 increased LH plasma concentration, however, it was less potent than Kp10 and its duration of action was even shorter^19^. Therefore, our results are the first to prove that it is possible with a single intramuscular injection of a Kp analog to synchronize ovulations that are fertile. For livestock management this has potentially immediate application. At present ovulation during the non-breeding season is obtained by a combination of progestogen priming and treatment with equine chorionic gonadotropin (eCG alias PMSG). However, the use of eCG comes with drawbacks: potential sanitary risk due to disease transmission (because eCG is extracted from blood serum), and immunological responses that affect the efficacy of ensuing treatments^20^. The replacement of eCG by Kp analogs is therefore foreseeable and it would be a major improvement in livestock management. In addition the highly synchronized ovulation induced by the analog may represent an advantage to improve the timing of artificial insemination.

Experiments in mice demonstrated that **C6** is also highly active in this species, and therefore likely to be usable in other mammals. The pattern of LH secretion triggered by **C6** in mice was different between sexes. In male the injection induced a rapid increase followed by a long-lasting plateau (more than 6 hours). Conversely, in female the pattern of LH plasma concentration was reminiscent of an LH surge. A possible explanation for this difference is an increase of estradiol negative feedback in females capable of reducing GnRH secretion, similar to what happens during the estrus cycle. Further studies would be necessary to explore this hypothesis and decipher the underpinning mechanisms.In juvenile mice KiSS1R is expressed in about 90% of GnRH neurons^21^. In prepubertal female rats icv injections of murine Kp10 every 12 hours for 5 days advanced puberty^7^ and in 25-day old rats human Kp54 injection after eCG priming induced ovulation^22^. In primates hourly pulses of Kp10 to juvenile male induced the pulsatile release of LH and FSH^6^. Data on Kp involvement in puberty onset in domestic animals is limited, but consistent with the hypothesis that an increase in Kp production and release are important elements^23^,^24^,^25^. All together these data corroborate the notion that kisspeptin system has a key role in puberty onset in mammals. In farm animals, establishment of puberty is the starting point of female’s lifetime productivity and methods have been developed to try to hasten it. In this respect, the demonstration that daily injections of **C6** advance puberty in mice is an important result that could translate into livestock management. Finally, delayed puberty is a rather common pediatric disorder^26^ and kisspeptin analogs might represent an interesting new therapeutic strategy.

Available data on repeated Kp administration are constrasted suggesting or not the induction of tachyphylaxis (see for example 4 and 6). We have shown that in prepubertal mice **C6** is still active after multiple injections, even though its efficacy is seriously affected. A decrease in response after multiple injections has a limited relevance when trying to induce ovulation in fertile females, because, as we showed, a single injection will suffice. Conversely, it is more relevant for treating pathologies requiring prolonged treatment. Desensitization of the receptor and/or GnRH/LH stock depletion are two non-exclusive hypotheses that could explain this phenomenon. Desensitization in response to an agonist is a phenomenon mainly depending on the dosing regimen. Further studies exploring different dosing would help to fine tune the treatment that better allows for continuous stimulation without desensitization. On the other hand, dosing regimen inducing a rapid desensitization could also find a therapeutic interest for treating hormone dependent cancer.

Recent data suggest that Kp could activate RFamide related peptide-3 (RFRP-3) receptors^27^,^28^,^29^,^30^, albeit with lower potency than KiSS1R. Inconsistent effects of RFRP-3 on LH secretion in different species (inhibitory, stimulatory or no effect) make it difficult to predict the possible impact of the activation of RFRP-3 receptors by Kp. However, the lack of effect on calcium mobilization by **C6** on cell line non-transfected with KiSS1R and on LH levels in KiSS1R KO mice indicates that KiSS1R is essential for **C6** stimulatory activity. KiSS1R is expressed in GnRH neurons^31^ and our electrophysiological data demonstrate that **C6** directly activates GnRH neurons in brain slices. Peptide-based compounds normally do not easily cross the blood brain barrier (BBB), questioning the possibility that peripheral injection of **C6** would directly stimulate the GnRH cell body. However, the introduction of a long-chain fatty acid increases lipophilicity, and ω-methylation of arginyl residue, by reducing hydrogen bonding, is also known to increase membrane permeability^32^. Thus, **C6**, which contains both these modifications, might be prone to cross the BBB. On the other hand, GnRH projections to the median eminence possess a unique mixture of dendritic and axonal properties^33^ and can respond to Kp to regulate GnRH secretion in a spike-independent manner^34^. Furthermore, it has been reported that GnRH terminals could release a significant amount of hormone, even in the absence of the neuronal cell body^34^,^35^,^36^,^37^,^38^. Hence, it is plausible that even though **C6** would not cross the BBB, its action on terminals may suffice to trigger GnRH release. Further studies will be necessary to test this hypothesis.

The preclinical results presented herein support the notion that in mammals it is possible to trigger and synchronize ovulation as well as to accelerate puberty using a kisspeptin analog. Hence, kisspeptin analogs hold the potential to address unmet needs in livestock management and might found applications also in human therapy.

## Materials and Methods

### Experimental animals

#### Ewes

Ile de France ewes (3 to 5 years old, weighing 65–90 kg) from INRA breeding facilities were maintained at the INRA research center (Nouzilly, France) under natural photoperiod. Experiments were carried out in accordance with existing national and international regulations (directive 2010/63/UE, Authorizations N° A 38801 and E37-175-2 of the French Ministry of Agriculture) and all procedures were approved by the local Animal Ethics Committee (authorization number 2012-03-7). Experiments were performed at the Unité Expérimentale PAO n°1297 (EU0028) of INRA, Centre Val de Loire.

#### Mice

All mice were obtained from the University of Otago animal breeding facility and were bred on a C57BL/6 J background, except for KiSS1R KO mice that were bred on a mixed 129S6/Sv/Ev background. They were group housed and maintained on a 12 h:12 h light:dark cycle at a constant temperature (21 ± 1 °C), with *ad libitum* access to a standard rodent diet containing 4.8% fat and 0% sucrose by weight (Specialty Feeds, Glenn Forrest, Western Australia) and water. The University of Otago Animal Ethics Committee approved all animal protocols. The estrous cycle stage of female mice was determined by daily vaginal smear, with mice being killed for electrophysiology experiments between 10 and 11 am.

All *in vivo* experiments were performed once.

### Peptide synthesis

Peptides **C1**–**C7** were assembled by standard automated Fmoc/*t*Bu solid phase peptide synthesis, except for the introduction of the triazole in **C2**–**C7**, the heterocycle being generated through solid-supported copper-catalyzed azide/alkyne cycloaddition as described earlier^11^. All the compounds were purified by reverse phase HPLC. See supplementary information for detailed procedures and full characterizations.

### Cell culture and screening assay

Receptor activation was monitored using Fluo4 NW Ca^2+^ assay kit in HEK293A cells transfected with the hKiSS1R as previously described^11^ (kind gift of professor N. de Roux, INSERM U690, Hôpital Robert Debré, Paris, France). Considering the type of analysis performed, assessment of second messenger activation of a heterologous expressed receptor, the use of potentially misidentified cells has no impact on the result. Basal fluorescence was measured with a plate reader (PolarStar Optima, BMG Labtech). Immediately after the basal reading, the test compound was added at different concentrations and the intracellular Ca^2+^ dynamic monitored. To generate concentration activity curves, the mean basal value was subtracted from the value obtained after stimulation. The area under the curve (AUC) was calculated and plotted against concentrations. Concentration activity data points were fitted to sigmoidal curves generated by GraphPad Prism 5 and EC_50_ automatically calculated.

### Testing of drugs in the ewe

To induce an artificial luteal phase intact cyclic (October–December) or anestrus acyclic (March–June) ewes were implanted with vaginal sponge containing FA (Chronogest, Intervet), a progesterone analog, for 14 days. To compare the activity of Kp, **C3**, **C5** and **C6** injection of test drug was performed during the treatment with FA. For the experiments of synchronization and induction of ovulation 24 hours before drug injection the vaginal sponge was removed and the same day a catheter (ID 1.0, L 52 mm, Intraflon 2, Vygon, France) was inserted in the jugular vein. On the day of experiment the test compound was injected intramuscularly and blood samples (2 mL) were taken at various time intervals post-injection: every10 minutes for the first hour, every 20 minutes from 2 to 6 hours, every 60 minutes from 6 to 10 hours and every 120 minutes from 10 hours to the end of the experiment). Data are expressed as means ± SEM (N = 6–12 per group, for details on group size see figure legends).

### Assessment of estrus behavior and ovulation fertility

Intact cyclic ewes (N = 10) treated as described above were individually presented to a sexually active ram six hours after **C6** injection. The presence or absence of typical estrus behaviors (i.e. immobilization with head lowered, looking over the shoulder as the ram nudges her flank or anogenital region) and mounting were recorded over a five minutes period. Afterwards, ewes (2–3 per group) were left overnight with a ram harnessed with a marking device to assess servicing. As an early marker of pregnancy a blood sample was collected 19 days post-injection and progesterone level measured by ELISA assay.

### Brain slice preparation and electrophysiology

Brain slice electrophysiology was undertaken as reported previously^39^. In brief, 250 μm-thick coronal brain slices were prepared from adult female C57BL/6 GnRH-GFP mice^40^ and cell-attached recordings (current clamp with zero holding current) of GnRH neurons were undertaken using a fixed-stage upright microscope under Nomarski differential interference contrast optics. Patch pipettes (3–5 MOhm) were filled with a pipette solution composed of (in mM) 145 NaCl, 3 KCl, 2.5 CaCl_2_, 10 HEPES, 1.2 MgCl_2_ (pH 7.35 adjusted by NaOH, ~290 mOsmol). Cells were exposed to 0.01. 0.1, 1, 10 and 40 nM concentrations of **C6** (stock solution of 0.5 mM prepared in DMSO giving final bath concentration of ~0.04% DMSO) through the bathing medium for 2–3 minutes periods. Signals were amplified with a Multiclamp 700B amplifier (CV7B; Molecular Devices, Foster City, CA) and sampled on-line (Digidata 1440 A interface; Molecular Devices) before being filtered (10 kHz for current clamp; Bessel filter of Multiclamp 700B) and digitized at a rate of 10 kHz for pClampex recording. Acquisition and subsequent analysis were performed with the Clampex 10 suite of software (Molecular Devices) and Origin Pro 7.5 (OriginLab Corporation, Northampton, MA, USA). Drug-induced changes in action potential (AP) frequency were determined as follows: Responses to **C6** were determined by sorting all APs into 10 s bins and generating histograms of AP frequency. The control period was considered to be the 3 minutes period immediately before drug administration. The response period was of variable duration (2–20 minutes) lasting from drug administration until the cell returned to baseline firing. If the increase in the average frequency of AP firing during the response period was >25% of the control period, the cell was considered to have responded to the drug.

## Pharmacodynamics of C6 effect on circulating LH concentration in wild type and KiSS1R null mice

Adult male and female KiSS1R KO mice^41^ were primed by 5 ip injections of 40 ng/100 μL GnRH at 30 minute intervals and an equivalent volume of saline was injected to wild type mice. This protocol allows the pituitary gland to become more responsive to GnRH in this mouse line as previously reported^41^. Blood samples (4 μL) were collected from the tail tip as previously described^42^, before the first, third and fifth injection, and 30, 105 and 125 minutes after the last injection. **C6** was injected immediately after the last blood sampling (0.3 nmoles/200 μL, a dose established in a preliminary experiment) and blood samples taken 20, 40, 60, 90, 120, 180, 300 minutes following injection. After 300 minutes a further injection of GnRH (40 ng/100 μL) was performed in KiSS1R KO mice, and a final blood sample was taken 30 minutes later to confirm the functionality of the HPG axis. Whole blood samples were collected in 150 μL of 0.1 M PBS-0.05% Tween20, mixed and moved to dry ice at the end of each sampling round. Experiments were conducted during the first 8 hours of the light phase to avoid any endogenous LH surges associated with the preovulatory stage of the female reproductive cycle. Data are expressed as means ± SEM (N = 4–7 per group, for details on group size see figure legends).

## Effect of C6 on pubertal timing in female mice

Twenty female mice, born synchronously by timed mating, were randomly assigned to daily treatment with either **C6** (0.15 nmol/100 μL) or saline only from postnatal day 26 to 30. They were monitored daily for indicators of pubertal onset, namely vaginal opening and then estrous cyclicity onset (first estrus, detected by vaginal lavage and cytology) until puberty had occurred in all animals. Puberty occurs around 4.5 weeks of age in female C57Bl/6 J mice in our animal facility. Blood samples to measure LH level were taken on the first and the last day of treatment.

## Hormone assays

A sandwich ELISA, adapted from Steyn *et al.*^42^, was used to measure LH concentration in mice. Monoclonal bovine LHβ 518 B7 (1:1,000 dilution) was used as the capture antibody (obtained from Lillian Sibley at University of California Davis), and the standard was NIDDK mouse LH reference preparation from AF Parlow (AFP5306A mouse RIA kit). The assay sensitivity was 0.2 ng/mL and the mean intra-assay coefficient of variation averaged 11%.

Plasma LH concentrations in ewe were measured using previously described radioimmunoassays^43^,^44^. The assay standard was 1055-CY-LH (equivalent to 2.2 NIH-LH-S1). Intra-assay coefficient of variation averaged 9% and assay sensitivity was 0.2 ± 0.05 ng/mL. Plasma FSH levels were measured using the reagents supplied by Tucker Endocrine Research Institute (Atlanta, Georgia, USA). Intra-assay and inter-assay coefficient of variation averaged 7.6% and 9% respectively; assay sensitivity was 0.1 ng/mL relative to the standard (Tuenere oFSH-std.1 equivalent to 1.0 NIH-FSH-S1). The cross reactivity with ovine LH was 0.03%.

Progesterone was measured by an ELISA assay on 96-well plate (Immuno Nunc Maxisorp C96) coated with goat anti-mouse IgG (Uptima UP462140, Interchim) overnight at 4 °C. After washing with Tris-Tween20 the plates were incubated with the secondary antibody (mouse monoclonal anti-progesterone, AbD, Serotec (10 μL in 16 mL Tris-BSA) together with 10 μL of samples overnight at 4 °C. The following day 50 μL/well of progesterone-alpha alkaline phosphatase conjugate (Immunometrics Ltd.)(10 μL P4-pal 6 mL Tris-BSA) was added to each well and incubated in the dark for 1 hour. Plates were washed with Tris-Tween20 and then were incubated with pNpp (Sigma-Aldrich) for about 2 hours at 37 °C and absorbance were recorded at 405 nm with a plate reader (TECAN). Intra-assay coefficient of variation averaged 8.5% and assay sensitivity was 0.25 ng/mL.

## Additional Information

**How to cite this article**: Decourt, C. *et al.* A synthetic kisspeptin analog that triggers ovulation and advances puberty. *Sci. Rep.* **6**, 26908; doi: 10.1038/srep26908 (2016).

## Supplementary Material

Supplementary Information
