# Supplementary material for: A synthetic kisspeptin analog that triggers ovulation and advances puberty
Source: Sci Rep. 2016 Jun 1;6:26908. doi: 10.1038/srep26908 (PMC4887910; doi:10.1038/srep26908)

Supplementary data

A synthetic kisspeptin analog that triggers ovulation and advances puberty

Decourt C., Robert V., Anger K., Galibert M., Madinier J.-B., Liu X., Dardente H., Lomet D., Delmas A.F., Caraty A., Herbison A., Anderson G., Aucagne V. Beltramo M.

Table of Contents

(I) PEPTIDE SYNTHESIS …………………………………………………………………….. 2

A- General information …………………………………………………………....... 2

B- General procedure for solid-phase peptide synthesis (SPPS) ……………………. 2

C- General procedure for triazole incorporation through solid-supported copper-catalyzed azide/alkyne cycloaddition …………………………………………….. 3

D- General procedure for peptide characterization and purification ………………... 3

E- Characterization of the KISS1R agonists **C1-C7** ……………………….……… 5

(II) Supplementary figures ………………………………………………………….....................................15

(I) PEPTIDE SYNTHESIS

A- General information

Unless stated otherwise, all reagents and anhydrous solvents were used without further purification. Protected amino acids, aminomethyl polystyrene, ChemMatrix resin, Rink linker and HCTU were purchased from Merck Biosciences (Nottingham, UK). Peptide synthesis grade DMF was obtained from Applied Biosystems (Courtaboeuf, France). Fmoc-TTDS-OH was obtained from Iris Biotech GmbH (Marktredwitz, Germany). Ultrapure water was prepared using a Milli-Q water system from Millipore (Molsheim, France). All other chemicals were purchased from Sigma Aldrich (St-Quentin-Fallavier, France) and solvents from SDS-Carlo Erba (Val de Reuil, France) and were used without any further purification. Polypropylene syringes fitted with polypropylene frits were obtained from Torviq (Niles, MI, USA) and were equipped with PTFE stopcocks from Chromoptic (Courtaboeuf, France).

B- General procedure for solid-phase peptide synthesis (SPPS)

SPPS was run on an automated synthesizer 433A from Applied Biosystem using Fmoc/*t*Bu chemistry at a 0.1 mmol scale using Rink amide ChemMatrix® as solid support. The elongation was carried out automatically using a 10-fold excess of the *N*-Fmoc amino acid (or hexadecanoic or acetic acid), 9.5-fold excess of the coupling agent HCTU and 20-fold excess of *N*,*N*-di-isopropylethylamine, in NMP or 1:2 NMP/CH_2Cl_2 in the case of hexadecanoic acid. The protecting groups used for the *N*-Fmoc amino acids were Arg(Pbf), Arg(Me,Pbf), Asn(Trt), Glu-O*t*Bu, Ser(*t*Bu), Trp(Boc), Tyr(*t*Bu) and Lys(Dde). Fmoc deprotection was performed using a 20% piperidine solution in NMP. The 0.1 mmol scale program purchased from the manufacturer was used, with a single coupling followed by capping with a 60-fold excess of acetic anhydride, 15.5-fold excess of *N*,*N*-di-isopropylethylamine and 1.8-fold

excess of hydroxybenzotriazole in NMP. Fmoc groups were cleaved with 20% piperidine in NMP.

The crude peptides were deprotected and released from the resin with TFA/H_2O/*i*Pr_3SiH/phenol, 87.5/5/2.5/5 for 2 h, and the peptide was precipitated by dilution into an ice-cold 1:1 diethyl ether/petroleum ether mixture, recovered by centrifugation and washed 3 times with diethyl ether.

C- General procedure for triazole incorporation through solid-supported copper-catalyzed azide/alkyne cycloaddition

The specific building blocks**,** (*S*)-2-azido-4-methylpentanoic acid (N_3Leu-OH) and *N*-Fmoc-propargylamine were synthesized as described earlier. (1)

N-Fmoc propargylamine (0.4 mmol, 4 equiv.) and CuBr.Me_2S (82 mg, 0.4 mmol, 4 equiv.) were dissolved in NMP (10 mL) under an argon atmosphere. After addition of *N*,*N*-di-isopropylethylamine (70 µL, 0.4 mmol, 4 equiv.), the mixture was transferred into a syringe fitted with a frit containing azidopeptide resin (0.1 mmol.) swollen in NMP. The green suspension was stirred by syringe rotation for 2 h at room temperature and the resin was washed successively with NMP, CH_2Cl_2, 1 M pyridine hydrochloride in 95:5 CH_2Cl_2/MeOH, CH_2Cl_2 and DMF. The reaction was monitored by UV titration of the Fmoc group cleavage at 301 nm (ε = *7800* cm^{-1} M^{-1}). In case of incomplete reaction, the cycloaddition protocol was repeated.

D- General procedure for peptide characterization and purification

HPLC analyses and purifications were carried out on a LaChrom Elite system consisting of a Hitachi L-2130 pump, a Hitachi L-2455 diode array detector and a Hitachi L-2200 autosampler. The machines were equipped with Nucleosil C18, 300 Å, 5 µm, 250 × 4.6 mm (flow rate: 1 mL/min) or Chromolith® High Resolution RP-18e, 150 Å, 100 x 4.6 mm (flow rate: 3 mL/min) for analyses and Nucleosil C18, 300 Å, 5 µm, 250 × 10 mm for purification

(flow rate: 3 mL/min). Solvents A and B were 0.1% TFA in H_2O and 0.1% TFA in MeCN, respectively. The purity of all peptides tested *in cellulo* was >95%, and >98% for those tested *in vivo* (analysis by C18 RP-HPLC).

MS analyses were performed on an Ultraflex MALDI-TOF instrument (Bruker Daltonics, Bremen, Germany) equipped with a 337 nm nitrogen laser and a gridless delayed extraction ion source. External calibration was accomplished using Flex-Control software (Bruker). The sample was co-crystallized with a solution of α-cyano-4-hydroxy-cinnamic acid (HCCA) as a matrix. The observed *m/z* corresponds to the monoisotopic ions unless stated otherwise.

E- Characterization of the KISS1R agonists

Compounds **1-4** have been previously described (1).

- **H-Tyr-Asn-Trp-Asn-Ser-Phe-Gly-Leu-Arg-Tyr-NH₂ (C1)** (Kp10)

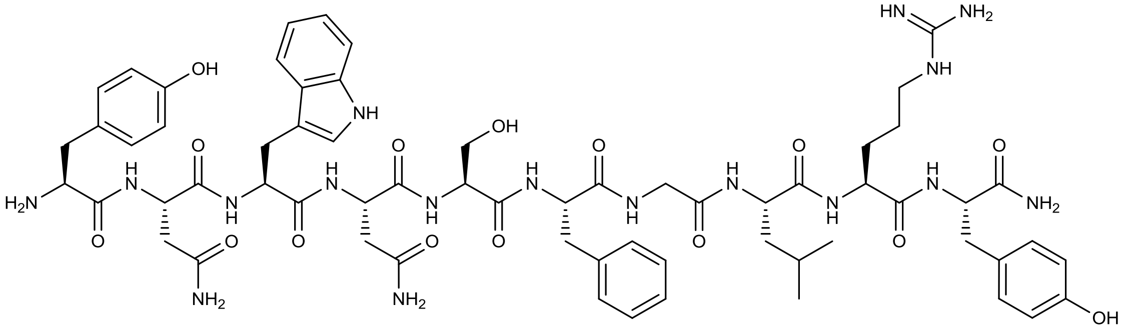

MALDI-TOF MS (*m/z*): $[M+H]^+$ calcd for $C_{63}H_{84}N_{17}O_{15}$: 1318.6, found: 1318.6.

HPLC (Nucleosil): analytical gradient 20-40% B/A over 30 min, retention time: 18.4 min.

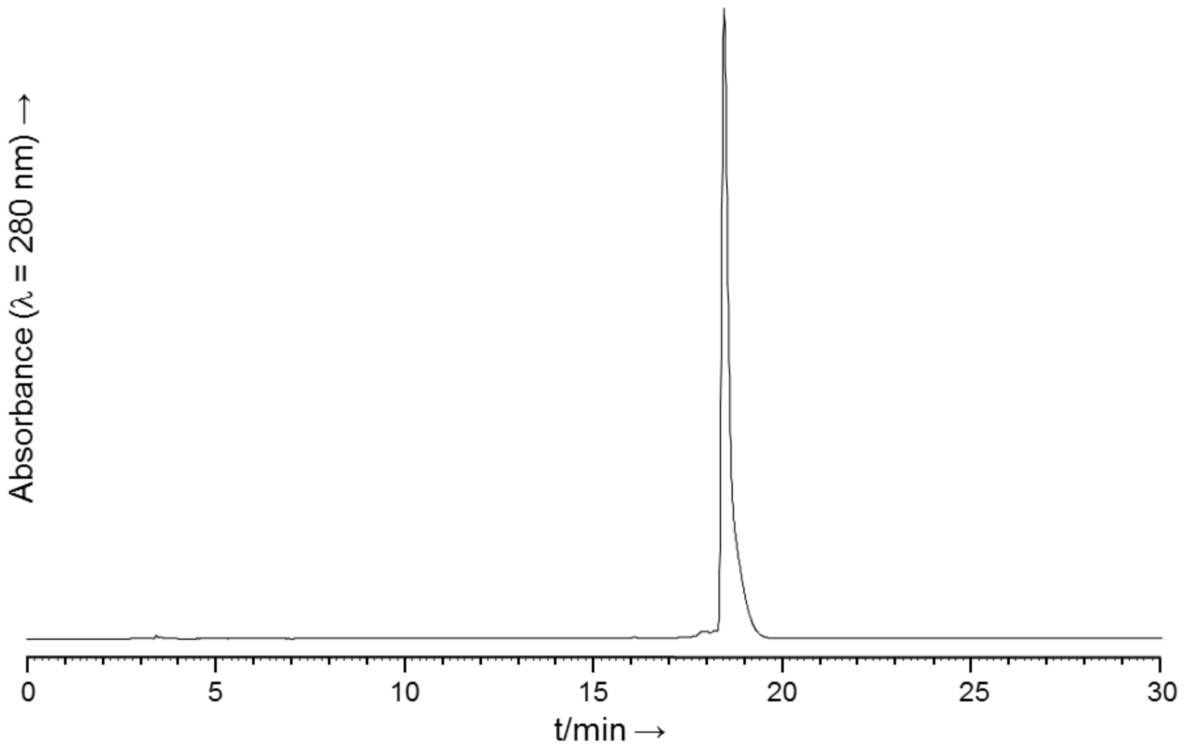

- **Ac-Tyr-Asn-Trp-Asn-Ser-Phe-GlyΨ[Tz]Leu-Arg-Tyr-NH_2 (C2)**

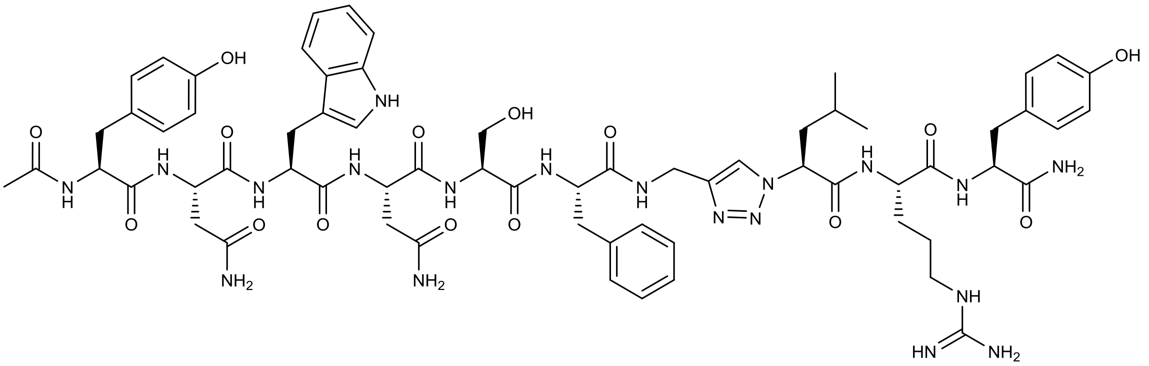

MALDI-TOF MS (*m/z*): $[M+H]^+$ calcd for $C_{66}H_{86}N_{19}O_{15}$: 1384.7, found: 1384.6.

HPLC (Nucleosil): analytical gradient 20-40% B/A over 30 min, retention time: 20.5 min.

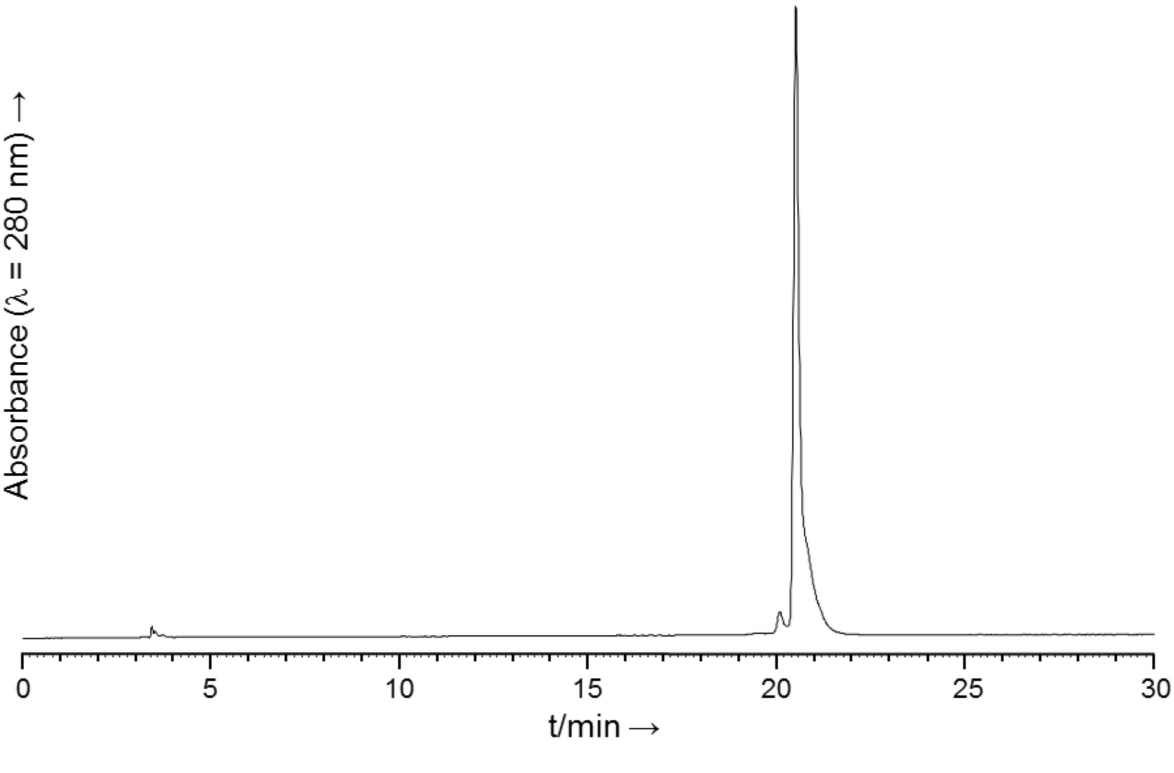

- **Ac-Tyr-Lys(-(*N*-hexadecanoyl-Glu-OH))-Trp-Asn-Ser-Phe-GlyΨ[Tz]Leu-Arg-Tyr-NH$_2$ (C3)**

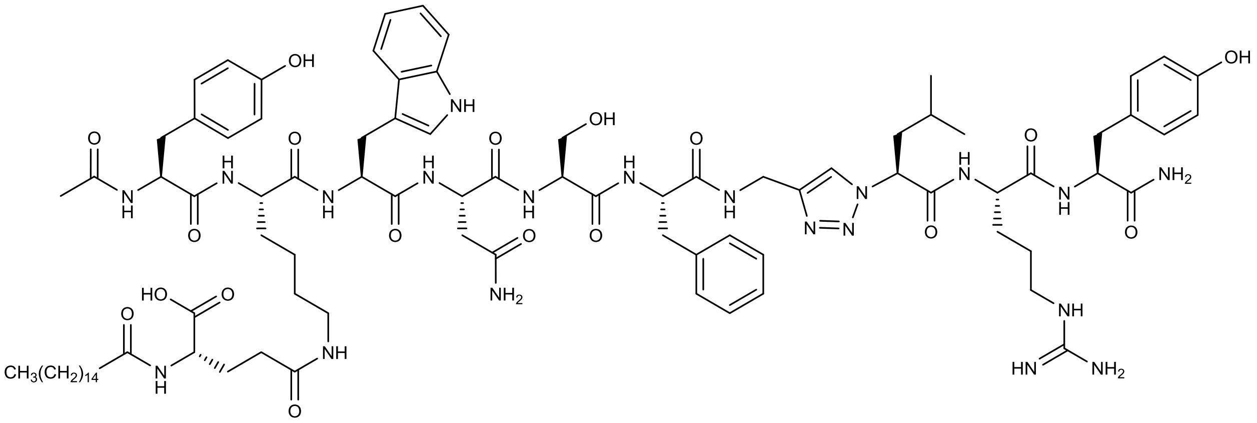

MALDI-TOF MS (*m/z*): $[M+H]^+$ calcd for $C_{89}H_{129}N_{20}O_{18}$: 1765.97, found: 1765.99.

HPLC (Nucleosil): analytical gradient 45-75% B/A over 30 min, retention time: 20.1 min.

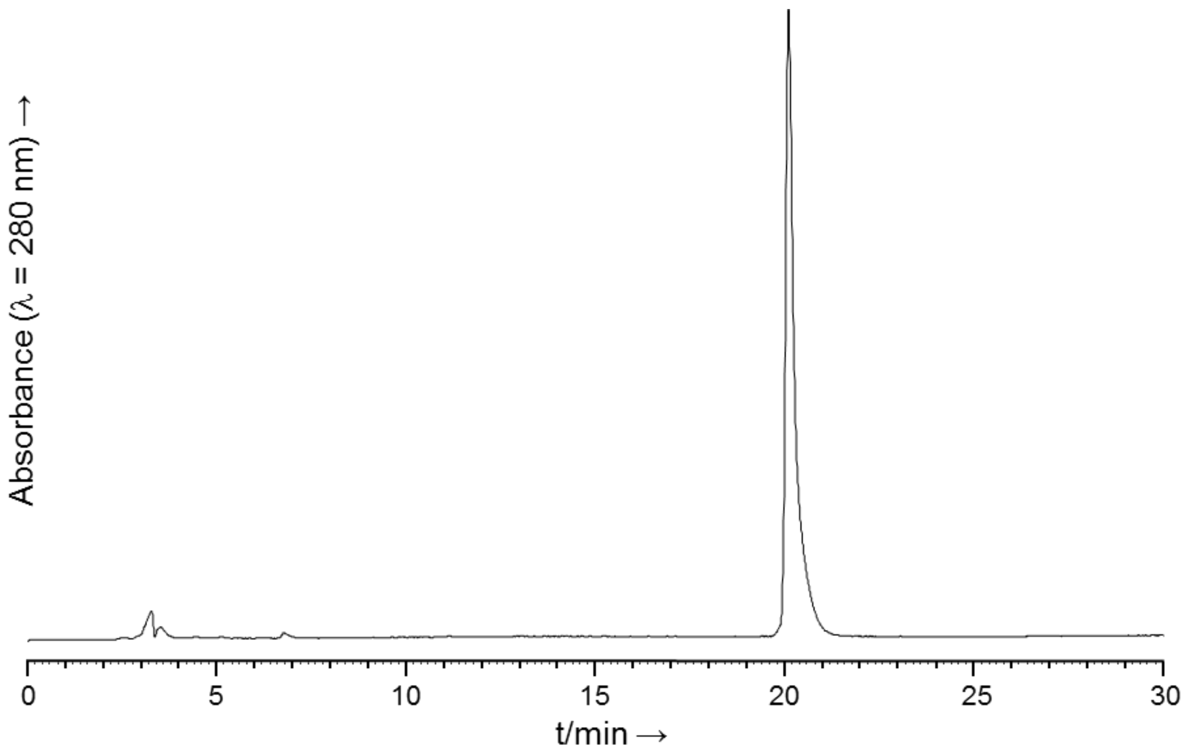
Absorbance (λ = 280 nm) →
0
5
10
15
20
25
30
t/min →

- **Ac-Tyr-Asn-Lys(□-(*N*-hexadecanoyl-Glu-OH))-Asn-Ser-Phe-GlyΨ[Tz]Leu-Arg-Tyr-NH$_2$ (C4)**

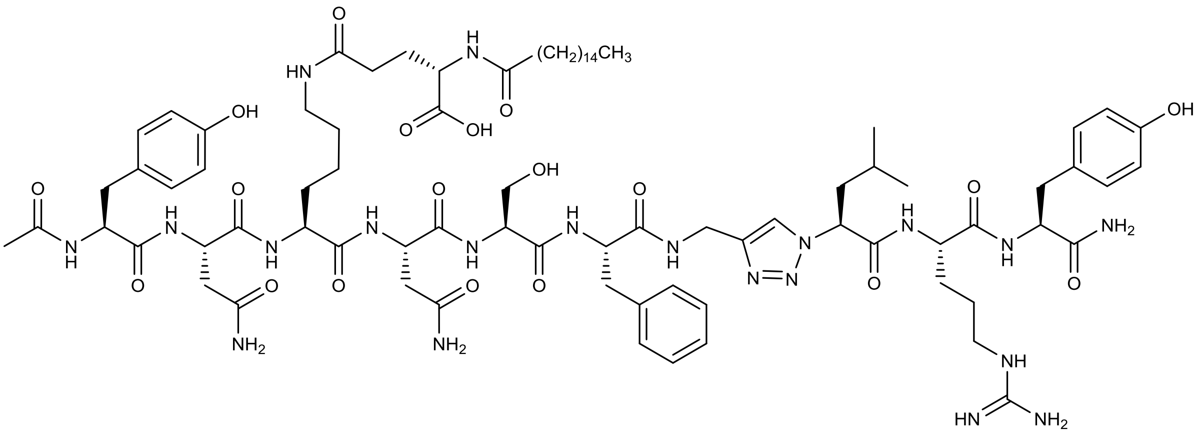

MALDI-TOF MS (*m/z*): $[M+H]^+$ calcd for $C_{82}H_{125}N_{20}O_{19}$: 1693.94, found: 1693.97.

HPLC (Chromolith): analytical gradient 45-75% B/A over 6 min, retention time: 3.02 min.

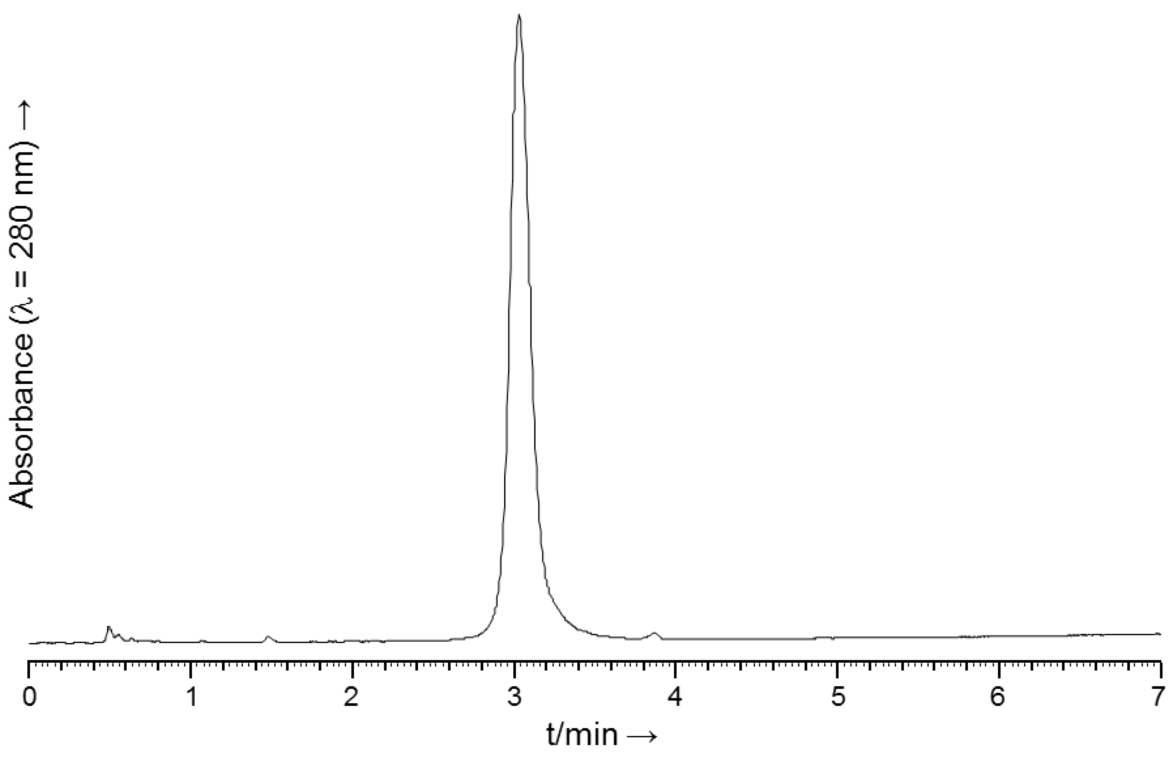

- **hexadecanoyl-□-Glu-Tyr-Asn-Trp-Asn-Ser-Phe-GlyΨ[Tz]Leu-Arg-Tyr-NH$_2$ (C5)**

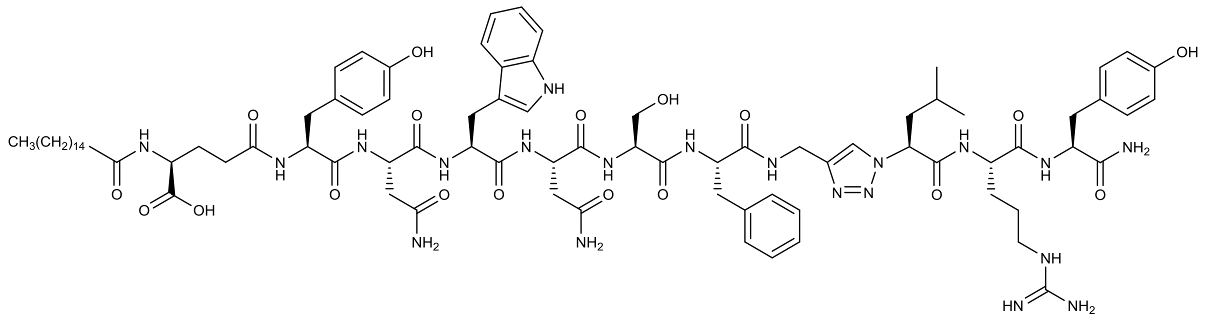

MALDI-TOF MS (*m/z*): [M+H]$^+$ calcd for $C_{85}H_{121}N_{20}O_{18}$: 1709.92, found: 1709.95.

HPLC (Chromolith): analytical gradient 45-75% B/A over 6 min, retention time: 4.03 min.

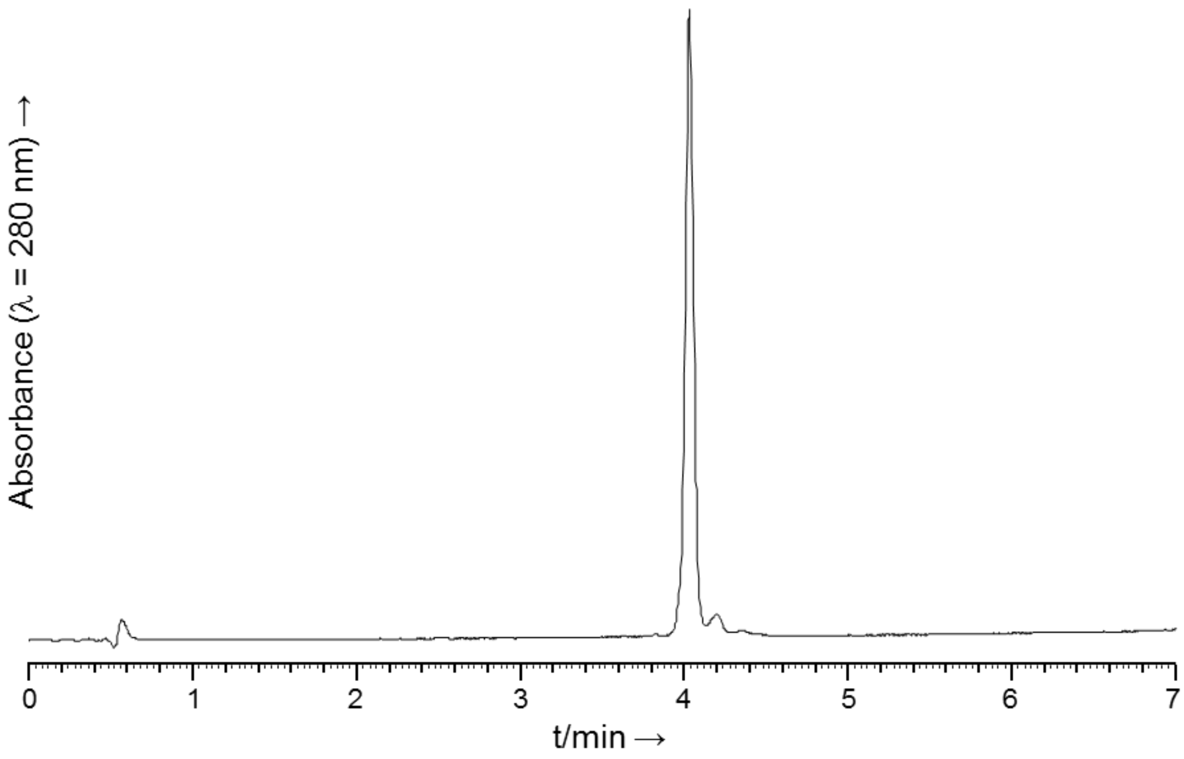
Absorbance (λ = 280 nm) →
0
1
2
3
4
5
6
7
t/min →

- **hexadecanoyl-□-Glu-Tyr-Asn-Trp-Asn-Ser-Phe-GlyΨ[Tz]Leu-Arg(Me)-Tyr-NH$_2$ (C6)**

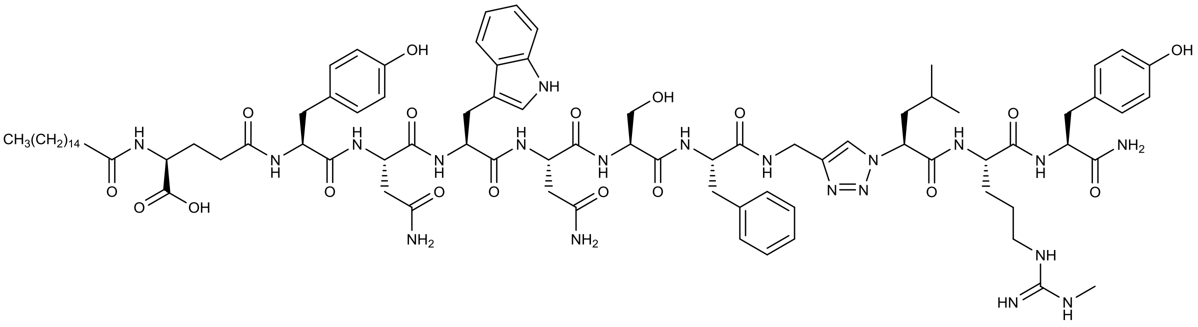

MALDI-TOF MS (*m/z*): $[M+H]^+$ calcd for $C_{86}H_{123}N_{20}O_{18}$: 1723.93, found: 1723.95.

HPLC (Chromolith): analytical gradient 45-75% B/A over 6 min, retention time: 3.80 min.

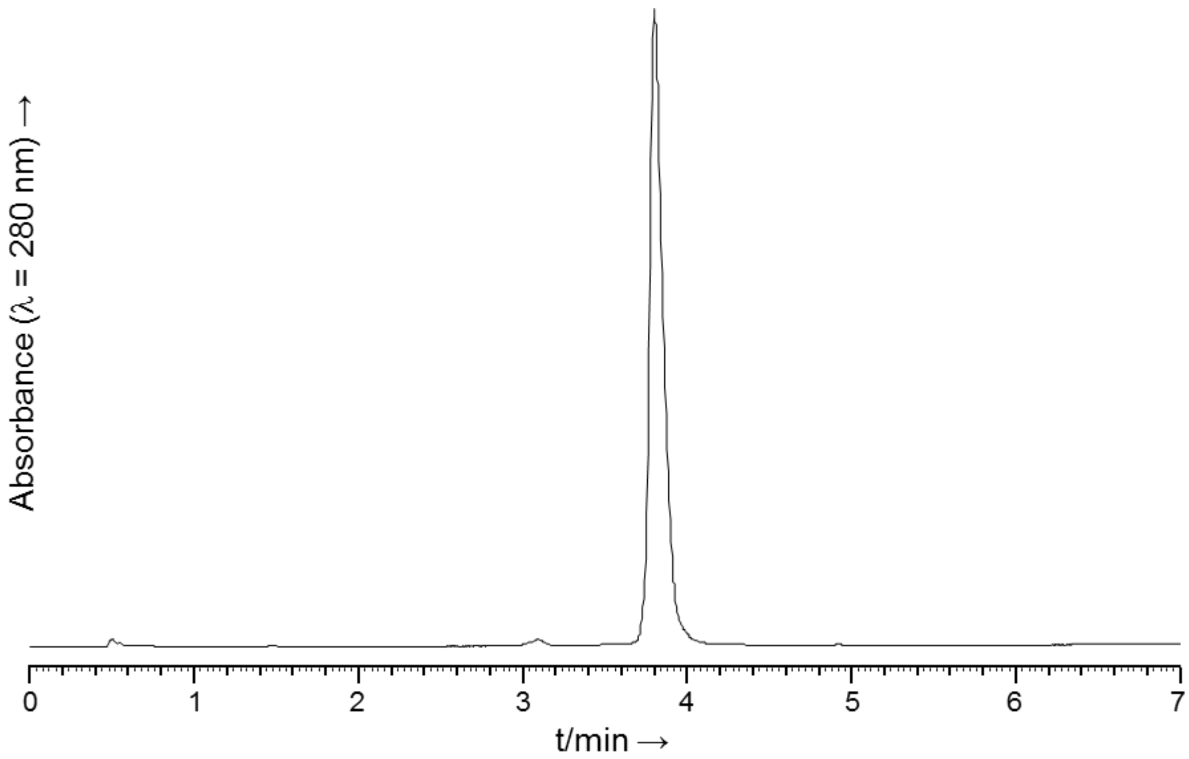

- **Ac-Tyr-Asn-Trp-Asn-Ser-Phe-GlyΨ[Tz]Leu-Arg(Me)-Tyr-NH$_2$ (C7)**

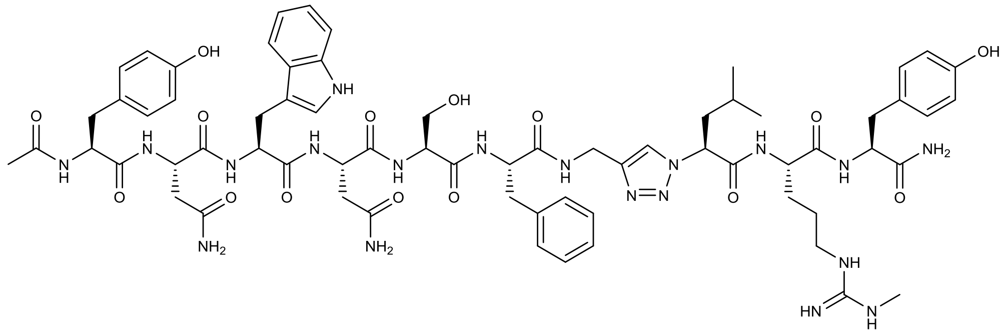

MALDI-TOF MS (*m/z*): $[M+H]^+$ calcd for $C_{67}H_{88}N_{19}O_{15}$: 1398.67, found: 1398.72.

HPLC (Nucleosil): analytical gradient 25-40% B/A over 30 min, retention time: 14.2 min.

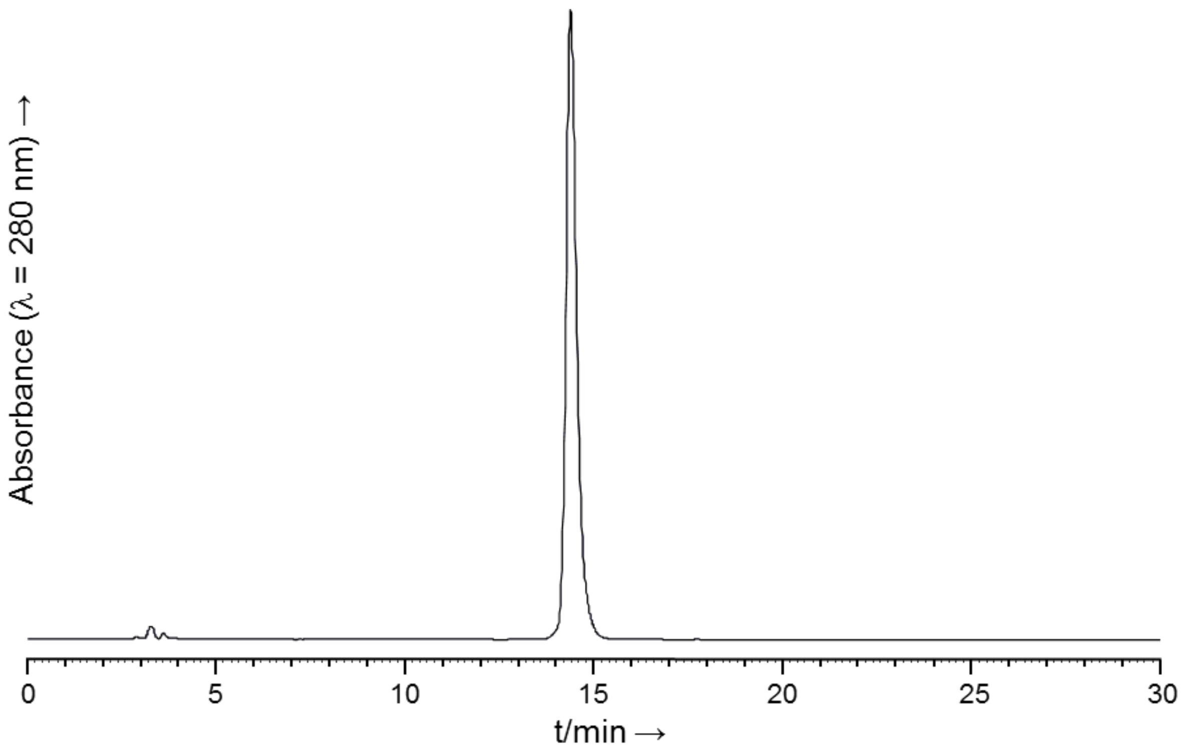

(1) Beltramo M, *et al.* (2015) Rational design of triazololipopeptides analogs of kisspeptin inducing a long-lasting increase of gonadotropins. *J. Med. Chem.* 58(8):3459-3470.

(II) Supplementary figures

Figure S1. Intramuscular injection of **C6** triggers ovulation in both breeding and non-breeding season. Individual profile of the LH plasma concentration of the ewes treated with 15 nmol/ewe of **C6** 24 hours after withdrawal of flugestone acetate (FA) during the non-breeding (N=12) or the breeding season (N=6). In both conditions (non-breeding and breeding season) **C6** induced a highly synchronous LH surge in all ewes (**A** and **C**). Individual profiles of progesterone blood level in both the non-breeding and breeding season (shown in **B** and **D**) confirm the formation of corpora lutea. ID number for each ewe is reported in the panel.

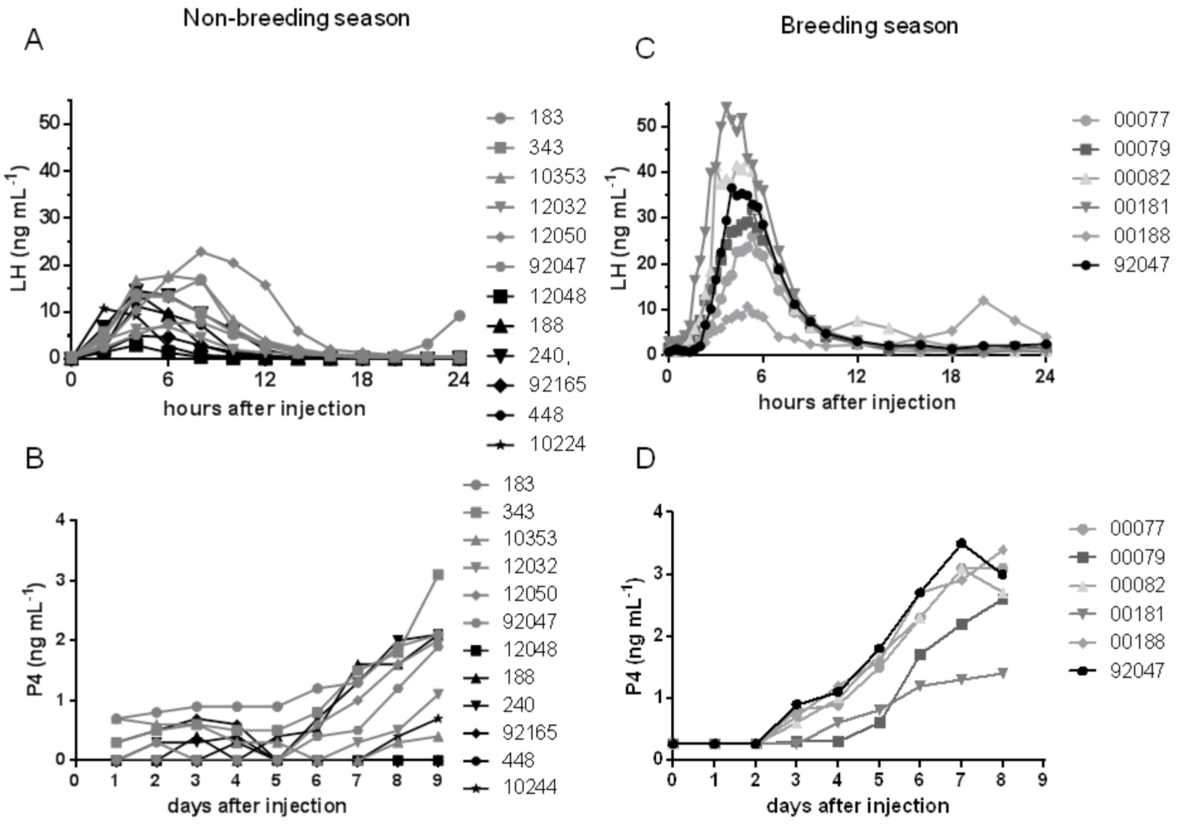

Figure S2. Intramuscular injection of **C6** increases FSH plasma concentration in the ewe. **C6** (15 nmoles) was injected intramuscularly at time zero and blood samples collected up to 48 hours post-injection. **C6** induced a biphasic increase of FSH with a first peak at about 5 hours. A second increase, starting around 18 hours post-injection, of lower amplitude but longer duration is also observed. This biphasic profile recalls the FSH secretion pattern of a natural estrous cycle in the ewe. Data represent the mean±SEM (N=6).

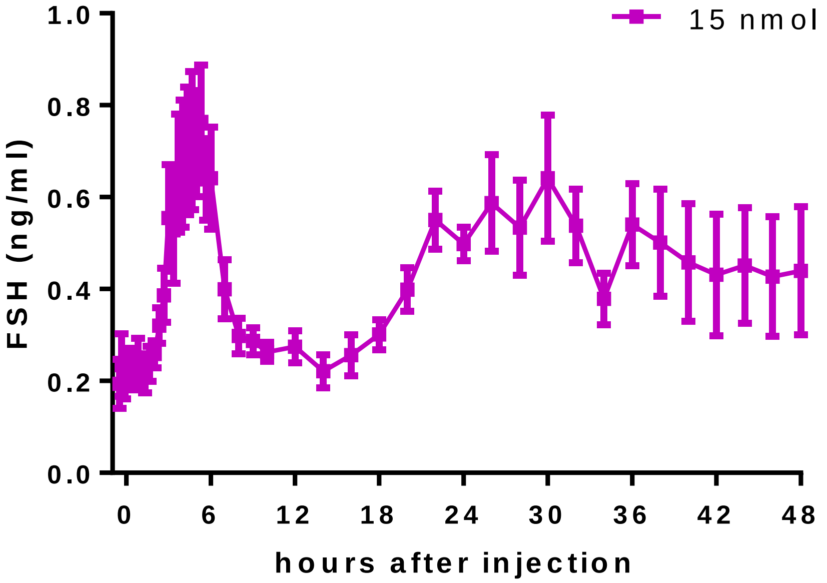

Figure S3. KiSS1R is required for C6-induced intracellular calcium mobilization. Concentration activity effect of C6 on intracellular calcium mobilization was assessed on HEK cells transfected with KiSS1R (blue square) and non-transfected parental cell line (purple triangle). In the absence of KiSS1R C6 has no effect on calcium mobilization confirming that its action is KiSS1R-mediated. Data represent the mean±SEM of 2 independent experiments performed in duplicate.

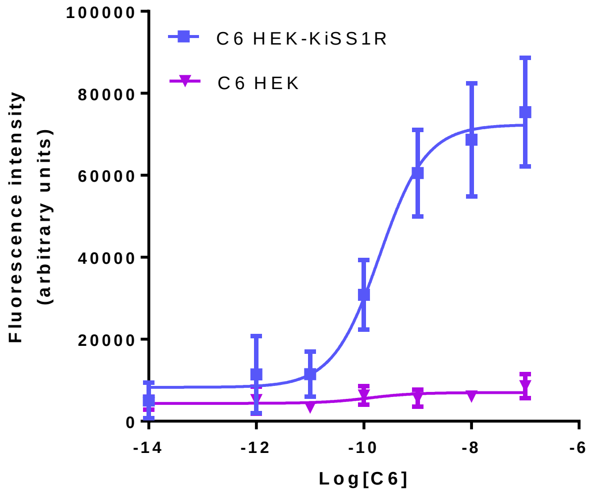

Figure S4. In mice compound 6 administration advances puberty. Plot showing the % of mice with vaginal opening (A) and day of first oestrus (B) after daily injection of compound **6** or control starting at P26 and ending at P30 (N=10).

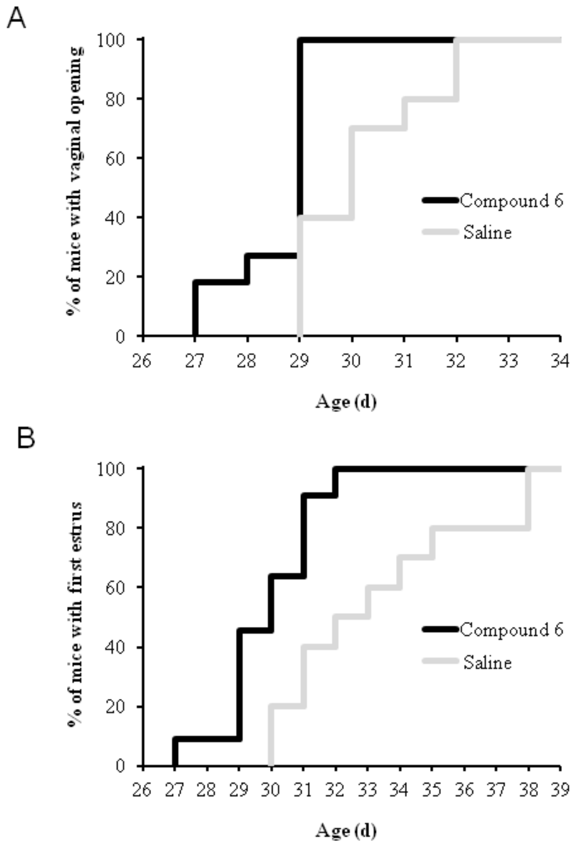

Figure S5. Following 5 daily dosing **C6** effect on LH secretion is reduced. **C6** was injected intraperitoneally once a day for five days and blood samples were taken 60 after the first and the last injection. The fifth injection of **C6** has a reduced effect on the increase of LH plasma concentration compared to the first injection (N=10). Statistic was performed by student T test ($P<0.001$ and $P<0.005$ for first and last injection respectively)

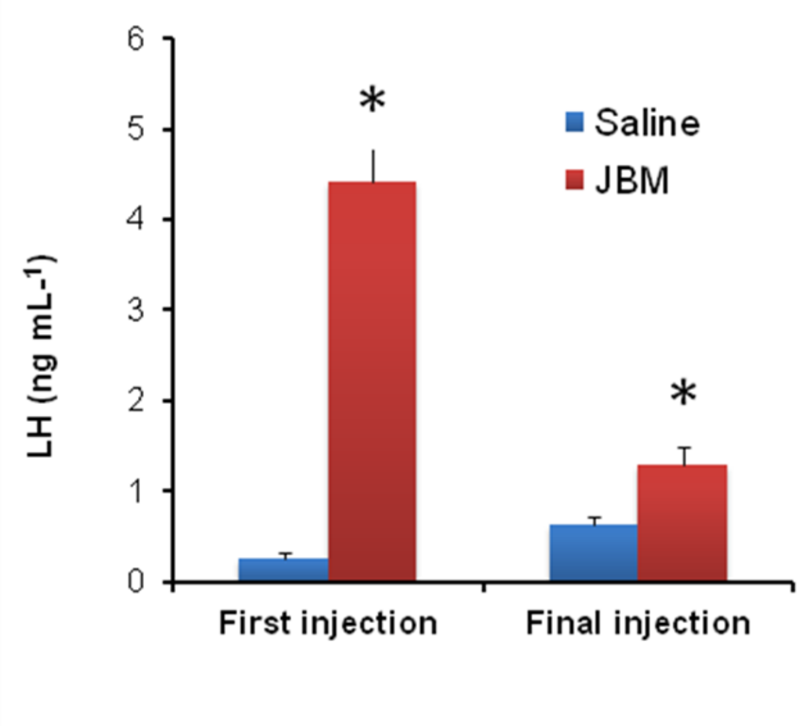

Supplement: Supplementary Information [file srep26908-s1.pdf]
